# Changes in the Chemical Composition of Polyethylene Terephthalate Under UV Radiation in Various Environmental Conditions

**DOI:** 10.21203/rs.3.rs-4402725/v1

**Published:** 2024-05-22

**Authors:** Sara Rostampour, Rachel Cook, Song Syun Jhang, YueJin Li, Chunlei Fan, Li-Piin Sung

**Affiliations:** 1 Infrastructure Materials Group, Materials and Structural Systems Division, National Institute of Standards and Technology, Gaithersburg, MD 20899, USA; 2 Bio Environmental Science Program, Morgan State University, Baltimore, MD 21251, USA

**Keywords:** Microplastics (MPs), Polyethylene Terephthalate (PET), NIST SPHERE, Fourier Transform Infrared Spectrometry (FTIR), Degradation, Chemical Change

## Abstract

Polyethylene terephthalate has been widely used in the packaging industry. Degraded PET micro-nano plastics could pose public health concerns following release into various environments. This study focuses on PET degradation under ultraviolet radiation using the NIST SPHERE facility at the National Institute of Standards and Technology in saturated humidity (i.e., ≥ 95 % relative humidity) and dry conditions (i.e., ≤ 5 % relative humidity) with varying temperatures (30 °C, 40 °C, and 50 °C) for up 20 days. ATR-FTIR was used to characterize the chemical composition change of degraded PET as a function of UV exposure time. The results showed that the cleavage of the ester bond at peak 1713 cm^−1^ and the formation of the carboxylic acid at peak 1685 cm^−1^ are significantly influenced by UV radiation. Furthermore, the formation of carboxylic acid was considerably higher at saturated humidity and 50 °C conditions compared to dry conditions. The ester bond cleavage was also more pronounced in saturated humidity conditions. The novelty of this study is to provide insights into the chemical degradation of PET under environmental conditions, including UV radiation, humidity, and temperature. The results can be used to develop strategies to reduce the environmental impact of plastic pollution.

## Introduction

Microplastics (MPs) are emerging contaminants that have been detected in various environments, and significant concerns have been raised over their potentially harmful effects on ecosystems and human health [1]–[3]. As one of the most widely used synthetic plastics globally, polyethylene terephthalate (PET) has been commonly used in many industries and consumer products due to its low cost, lightweight nature, durability, and high transparency [4]–[7]. PET ranks fourth among commonly used packaging plastics, raising concerns about the overall plastic footprint and potential environmental impact [8]. Some of the significant applications of PET include plastic bottles and containers, synthetic textile fibers, food and beverage packaging, electronics, automotive parts, and more [6], [9]–[12]. The versatility of PET has led to massive production volumes, with an estimated annual usage of over 30 million tons worldwide [13].

However, the growing utilization of PET and improper disposal have led to significant plastic pollution, which has environmental and human health risks. For example, weathering and degradation of PET can release microplastic particles (< 5 mm) and associated chemical contaminants into ecosystems [14]–[17]. PET microplastics have been detected across terrestrial and marine habitats, leading to ingestion and bioaccumulation through the food chain [18]–[23]. In addition, studies have suggested that ingesting or accumulating these PET degradation microplastics may cause inflammatory, carcinogenic, mutagenic, or endocrine-disrupting effects in organisms [24], [25]. PET’s degradation can lead to releasing its constituent monomers, such as terephthalic acid, ethylene glycol, and other additive chemicals that have raised toxicological concerns [16], [26]. Furthermore, the long-term toxicological impacts of persistent exposure to microplastics and chemical additives from degraded PET in the environment remain largely unexplored. Understanding the environmental degradation pathways of PET is crucial to assessing such ecosystem and human health risks.

PET is a semi-crystalline polyester formed by the polymerization of ethylene glycol and terephthalic acid [27] and can persist in the environment for hundreds of years due to its high resistance to degradation [28], [29]. It is well established that solar ultraviolet (UV) radiation facilitates the fragmentation of plastic litter in land and aquatic environments [30], [31]. Prior studies have examined the photodegradation of PET under UV radiation and characterized the resulting chemical changes. Radiation with UV-B wavelengths (280 nm to 315 nm) has been found to cause the highest degree of PET degradation [32]–[34]. The primary photochemical reactions include chain scission of the ester bonds in the PET backbone, leading to reduced molecular weight and oxidation, forming carbonyl and carboxyl groups [35]–[38]. The ester carbonyl stretching vibration in the PET backbone structure appears at a wavenumber at approximately 1713 cm^−1^ in the mid-range Fourier transform infrared spectroscopy (FTIR) spectrum [39]–[41]. The intensity of this peak at 1713 cm^−1^ can be used to track the extent of ester bond cleavage during UV exposure. A declining intensity of the absorbance band at 1713 cm^−1^ indicates that the PET polymer chains are undergoing scission at the ester linkages due to photochemical reactions [41], [42]. Also, the formation of carboxylic acid photoproducts from PET degradation appears as a peak at approximately 1685 cm^−1^ in the mid-range FTIR spectra. An increase in the intensity of the absorbance band at this wavelength indicates the formation of carbonyl-containing degradation products, which can be used to track the extent of photooxidative PET degradation during UV exposure [39], [41], [43], [44].

Several studies have examined how factors like temperature, humidity, and intensity of UV radiation affect PET degradation rates and mechanisms. Elevated temperatures above the PET glass transition temperature enhanced photodegradation by increasing chain mobility and oxygen diffusion [15], [30], [45]. In another study, it was demonstrated that higher exposure temperatures could accelerate the reduction of high-density polyethylene’s (HDPE) ductility within the applied temperature range [46], and a 10 °C increase in temperature results in a 20 % to 30 % increase in degradation rate [47]. Higher humidity accelerates PET photodegradation through increased moisture penetration and oxidation [48]. Humidity could cause oxidation of the aromatic ring and the formation of small molecules that readily sublime or are washed away [47]. Furthermore, the effects of humidity on different plastic materials are complex and depend on other factors, such as temperature and UV radiation [47]. The degree of UV exposure also directly controls the extent of bond scission and photooxidation reactions. The intensity and wavelength of UV radiation can affect the rate of degradation, leading to changes in color and gloss loss for several types of plastic materials [15], [46], [49], [50]. Nevertheless, the influence of UV radiation on weathering processes can be complex and often interacts with other factors such as temperature and humidity. Most previous photodegradation studies of PET or other plastic materials have focused on limited exposure conditions (e.g., a single temperature or humidity), which leaves gaps in our understanding of how these environmental factors interact to influence the chemical changes in PET under UV radiation.

This study’s novelty lies in systematically investigating how UV radiation, humidity, and temperature impact the chemical composition of polyethylene terephthalate (PET) under controlled conditions that mimic real-world environments. The FTIR detected the PET chemical functional group changes during UV radiation. The study’s results offer valuable information on how PET degrades under different environmental conditions. These findings can help develop effective strategies to reduce the environmental impact of plastic pollution, which is essential for mitigating its ecological risks.

## Method and Materials

### Sample Preparation

**Fig. 1:**
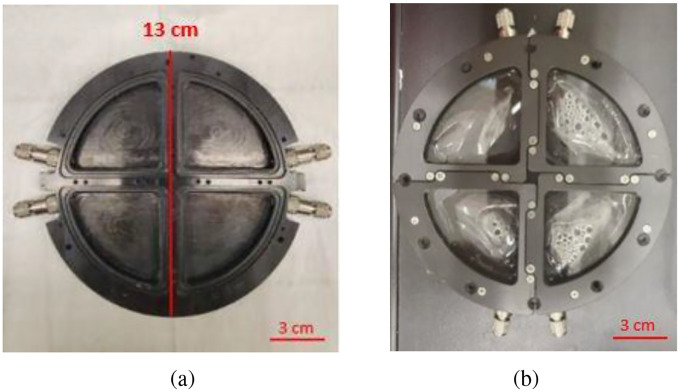
PIE cells (a) without and (b) with samples sealed by glass in a NIST SPHERE chamber under controlled environmental conditions

PET samples were prepared from common brands of water bottles. The bottles’ upper flat part (just below the cap) was cut into four samples, each 3 cm to 4 cm in length and 2 cm to 3 cm in width. Before UV exposure, the PET samples were washed with ethanol (Sigma-Aldrich, 100%) to remove surface contaminants. The PET samples were weighed with an analytical balance (METTER TOLEDO model, AB265-S/FACT), and their thickness was also measured with a caliper (SPI/13–610-1 model) from ten different locations of the specimen to reach an average thickness. No change in the mass (average = 0.24 g, n = 60, Standard Deviation (SD) = 0.03) and thickness of samples (average = 0.19 mm, n = 60, SD = 0.03) was observed after the experiment. After the pre-processing, PET samples were inserted into the Photochemical Internal Environment (PIE) cell for a UV exposure experiment. This is the first time this device was used for this type of experiment. The PIE cell (Fig. 1) is a specialized chamber that studies photochemical reactions under controlled environmental conditions. The PIE cell is made of aluminum (black anodized) with a diameter of 13 cm. Each PIE cell has four chambers sealed by quartz glasses that expose the individual PET samples to different humidity levels. To achieve saturated humidity, deionized water (DI water), provided by NIST, is injected into the cells using a syringe.

### UV Exposures

UV exposure experiments were performed using the NIST-developed SPHERE (Simulated Photodegradation via High Energy Radiant Exposure) in Gaithersburg, Maryland [46], [47]. The SPHERE is a 2 m diameter integrating sphere illuminated with six microwave-driven, electrodeless metal halide lamps similar to D-type UV curing lamps. The experiment employed a factorial experimental design to investigate the effects of UV intensity, humidity, and temperature on the photodegradation of PET samples. The UV intensity was set at the full-intensity UV (≈ 160 W/m^2^, integral intensities from 295 nm to 400 nm, daily dose ≈ 13.8 MJ/m^2^) for all experiments [47]. Two levels of humidity were tested: dry conditions (i.e., ≤ 5 % relative humidity) and saturated humidity (i.e., ≥ 95 % relative humidity). Additionally, the PET samples were exposed to three different temperature levels, 30 °C, 40 °C, and 50 °C, to explore the effects of temperature on PET photodegradation. For each exposure condition, the PET samples were placed in a photochemical internal environment (PIE) sample holder in the SHPERE chambers and exposed to UV radiation for up to 20 days. (20 days’ exposure on the SPHERE ≈, one year of sunlight exposure in South Florida, and 280 MJ/m2 in the same spectral range). Samples were removed from the PIE cells every two days after the initial exposure for chemical characterization. This allowed for monitoring chemical changes in the PET samples over the 20-day exposure period.

### Chemical Characterization by Fourier-transform Infrared (FTIR) Spectroscopy Measurements

Chemical changes in the PET samples were characterized via Attenuated total reflectance-Fourier transform infrared (ATR-FTIR) spectroscopy (Thermo Scientific, Nicolet iS50) [51], similar to methods employed by [52]–[56]. FTIR has often been used to quantify the carbonyl content in oxidized polymers, using both transmission and ATR techniques to establish oxidation extent and reaction kinetics for degradation comparisons and modeling [57]. Before ATR-FTIR measurements, the sample is placed onto the ATR crystal and pressed down using a swivel press to ensure optimal contact between the sample and the crystal. The measurements were carried out at least three locations for the inner (unexposed) and outer (exposed) surfaces, respectively, of the PET samples. Due to the limited space of the PIE cell, two replicas were used for each exposure time point at various exposure conditions. To gain a better estimate of measurement uncertainty in FTIR-ATR, a series of parallel experiments was conducted using ten PET samples in the same exposure conditions using regular sample holders, as mentioned [46]. The spectral range was set from 400 cm^−1^ to 4000 cm^−1^ with a measurement depth of approximately 2 mm using a diamond ATR crystal. The FTIR spectrum of PET was characterized by specific bands corresponding to various functional groups; this is further discussed in the section on data analysis. After baseline correction, all spectra were normalized to a particular band mostly unaffected by degradation processes at the peak of 1410 cm^−1^, as suggested by previous studies [36], [42], [58].

### Data Analysis

The absorbance level of sensitive bands, such as ester bonds, changes during the degradation process, while the absorbance level of insensitive bands remains constant throughout environmental aging. Therefore, an insensitive band should be used to normalize the absorbance of the sensitive band, as it does not exhibit any tendencies with aging time. The band at a wavelength of 1410 cm^−1^, attributed to the in-plane ring mode, is unaffected (insensitive) by environmental factors [9], [36], [42], [43], [59]. Thus, it is an excellent reference for normalizing spectral intensities among polymers. Variations in IR absorption bands were used to monitor the cleavage of ester groups in the main chain at wavenumber 1713 cm^−1^, representing carbonyl C=O stretching and being one of the most prominent absorption peaks in FTIR spectra [36]. Additionally, the formation of carboxyl groups was detected in polymers at the wavenumber of 1685 cm^−1^, indicating the presence of CO, CO_2_, and -COOH (carboxylic acid end groups), which are the primary photodegradation products in PET [34], [42], [43], [60].

The final IR spectra were exported from Omnic software (Thermo Scientific) and imported into Origin Software (Origin Lab Corporation) for baseline correction to facilitate qualitative analysis. Subsequently, the spectra were normalized at the peak at 1410 cm^−1^ to investigate chemical changes of the peaks at 1713 cm^−1^ (indicative of ester bond cleavage) and 1685 cm^−1^ (indicating the formation of carboxylic acid) in various environmental conditions over different time periods, ranging from a few days to 20 days. Multiple indices were calculated from the normalized FTIR spectra using a linear, end-to-end baseline. These included the vinyl index (peak area integration from 930 cm^−1^ to 880 cm^−1^), carbonyl index (integration from 1850 cm^−1^ to 1650 cm^−1^), and hydroxyl index (integration from 3575 cm^−1^ to 3125 cm^−1^), among others [46], [61]. This area-integrated approach was preferred over the more commonly employed peak intensity approach, as it accounts for the different functional groups contributing to the main bands. Since the *Carbonyl Index* can be a valuable indicator for quantifying the extent of PET degradation over time [41], it was calculated by measuring the absorbance at the peak of 1713 cm^−1^ divided by the absorbance at the peak of 1685 cm^−1^ (ester/acid ratio) for the analysis of hydrolysis characterization (Eq. 1).

(Eq. 1) $$\text{Carbonyl}\;\text{Index}=\frac{\text{cleavage}\;\text{of}\;\text{ester}\;\text{bond}\;\text{at}\;\text{peak}\;1713{\;\text{cm}}^{-1}}{\text{formation}\;\text{of}\;\text{carboxylic}\;\text{acid}\;\text{at}\;\text{peak}\;1685{\;\text{cm}}^{-1}}$$

It’s important to acknowledge that all measurements have inherent uncertainty. This reflects the limitations of the measuring instrument or process itself. This study’s error bars represent one standard deviation (SD). The SD calculated from three measurements on the same sample (n = 3) in the PIE cell was ≈ 6.2 %, and the SD for a set of 10 samples (n = 30, with three measurements on each sample) exposed to identical conditions was ≈ 6 %. The measurement uncertainties reported in this paper were obtained by calculating the SDs from a total of 33 measurements (n = 33), and the SD value was approximately 6 %. Data points indicate the mean, and the error bars present the standard deviation values (n = 33).

### Deconvolution

**Fig. 2:**
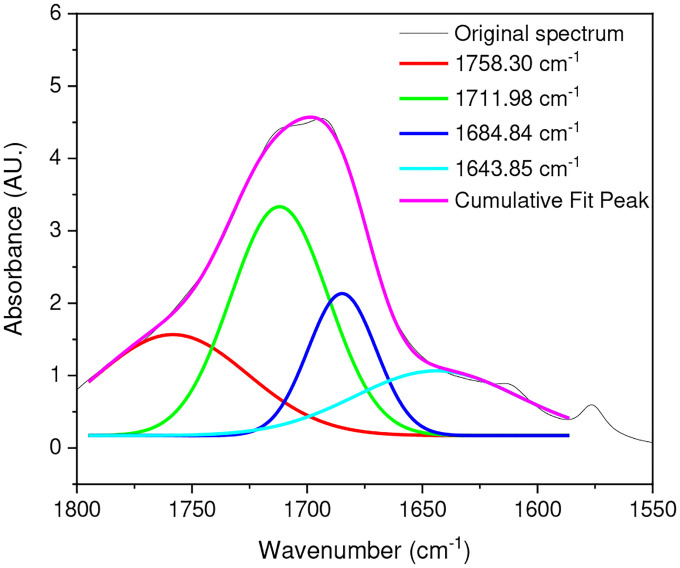
The peak-resolving results in the carbonyl region of PET aged for 20 days at 50°C in dry conditions

The deconvolution of a composite peak into its individual components is crucial for understanding various types of spectra, including FTIR spectra. Peak-resolving, a technique that relies on the second derivative, is an effective method for distinguishing and resolving overlapping absorbance [41], [45]. This technique is particularly valuable for distinguishing between different carboxylic and ester groups within the carbonyl region, as illustrated in Fig. 2.

Fig. 2 reveals that the original spectrum comprises four closely situated peaks at (1758, 1711, 1684, and 1643) cm^−1^. Among these, the peaks at 1685 cm^−1^ and 1643 cm^−1^ may originate from acid-terminated hydrolysis products, while the peaks at 1758 cm^−1^ and 1711 cm^−1^ may represent the absorbance of esters within hydrolysis products in the main chain. The combined areas of the former and latter peaks reflect the total quantity of acids and esters in the cumulative fitted peaks. In the carbonyl region of an IR spectrum of PET, various carbonyl species overlap, primarily esters and acids derived from terminal carboxyl groups. During the hydrothermal aging of PET, hydrolysis results in the formation of carboxylic and hydroxyl end groups. Consequently, the concentration of carboxylic groups increases with aging time, while that of ester groups decreases. The two peaks at 1711 cm^−1^ and 1758 cm^−1^ can be attributed to the ester group within the main chain, the end carboxyl group, and their hydrogenbonded associations.

## Results and Discussion

### Effect of UV Radiation

**Fig. 3:**
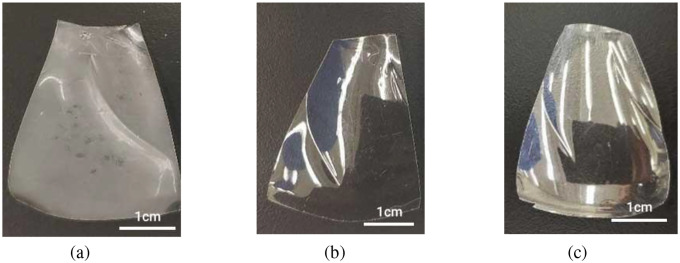
PET samples (a) after 20 days of exposure to 40 °C in saturated humidity, (b) in dry conditions, and (c) before treatment

The chemical properties of PET are essential for specific intended applications, and detecting changes in PET’s chemical composition helps us understand its degradation pathway. Initially, PET is ductile and transparent, but it becomes cloudy, yellow, and brittle after degradation [62], [63] (Fig. 3), while the PET sample in the dry condition is still transparent and soft after 20 days of aging, and there is no noticeable difference in appearance between the dry and untreated samples. These changes result in cracking and surface erosion due to oxidative degradation in the amorphous and crystalline components [64]–[66].

**Fig. 4:**
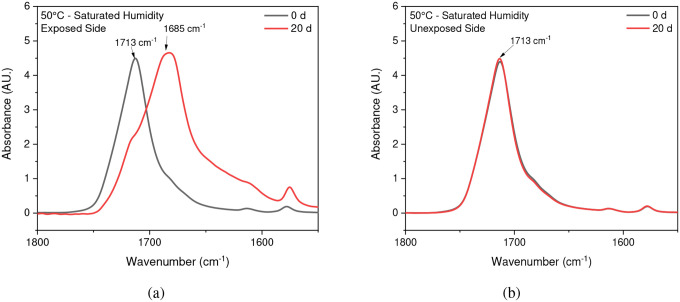
ATR-FTIR results of (a) exposed and (b) unexposed surfaces at 20-day exposure at 50 °C/saturated humidity. The arrows indicate the location 1713 cm ^−1^ and 1685 cm ^−1^ IR band

Chain scission is one of the primary outcomes of PET photodegradation, leading to increased crystallization and the formation of various degradation products. ATR-FTIR was used to identify and quantify surface crystallinity and degradation products, providing a better understanding of how different environmental conditions influence changes in chemical composition. As mentioned in the introduction section, chain scission in PET occurs in the vinyl-ester bond at 1713 cm^−1^, and one of the degradation products is the confirmation of carboxylic acid at 1685 cm^−1^ [42], [67]. The chemical changes are evident on the surface exposed to UV radiation. In contrast, no chemical changes are observed on the inner surface or the surface not exposed to UV radiation, as illustrated in Fig. 4. Nevertheless, other studies have indicated that the mid-range IR spectra of PET bottle’s inner and outer surfaces exhibit similar results, suggesting that degradation occurs on both the outside and inside of the bottle under natural marine environment conditions [51], [68]. However, the degradation process depends on the life cycle of PET bottles in nature. The most significant cleavage of the ester bond occurs under full-intensity UV radiation, saturated humidity (i.e., ≥ 95 % relative humidity), and 50 °C conditions. Fig. 4 illustrates a notable distinction between the outer (exposed) and inner (unexposed) surfaces of the PET pieces concerning ester bond cleavage at 1713 cm^−1^ and the presence of carboxylic acid at 1685 cm^−1^.

### Effect of Saturated Humidity

**Fig. 5:**
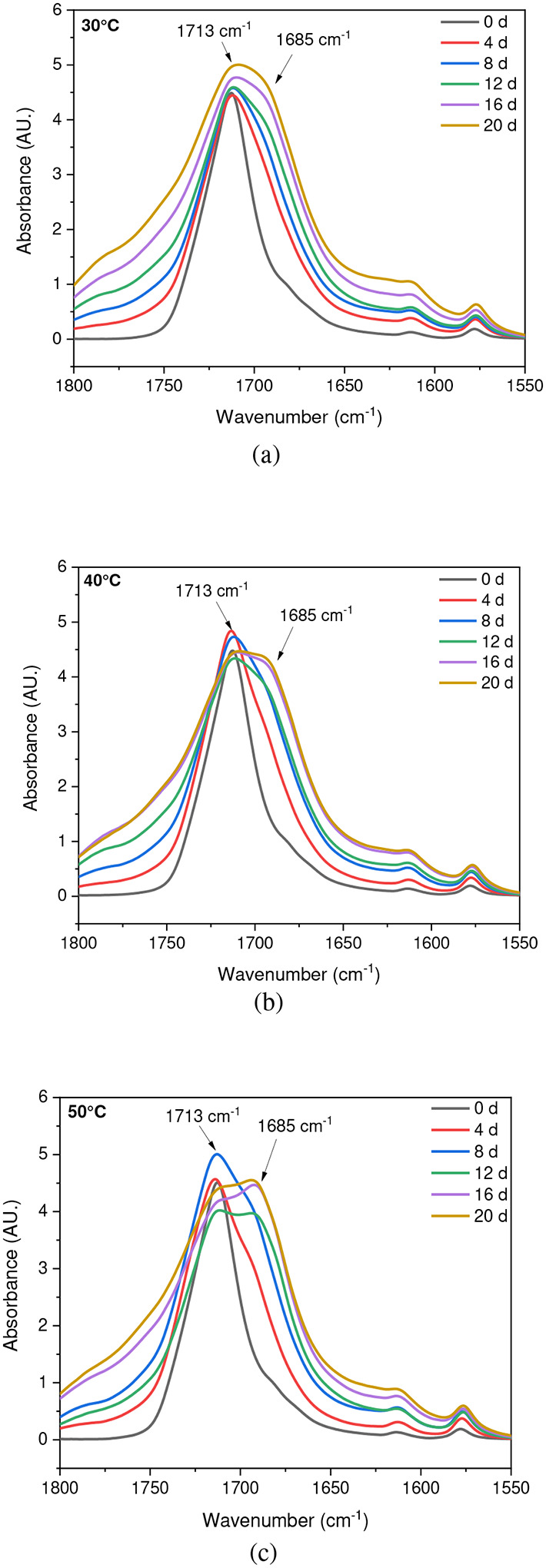
ATR-FTIR results of UV-exposed outer surfaces in dry conditions at (a) 30 °C, (b) 40 °C, and (c) 50 °C. The arrows indicate the location of 1713 cm ^−1^ and 1685 cm^−1^ IR bands in the spectrum

**Fig. 6:**
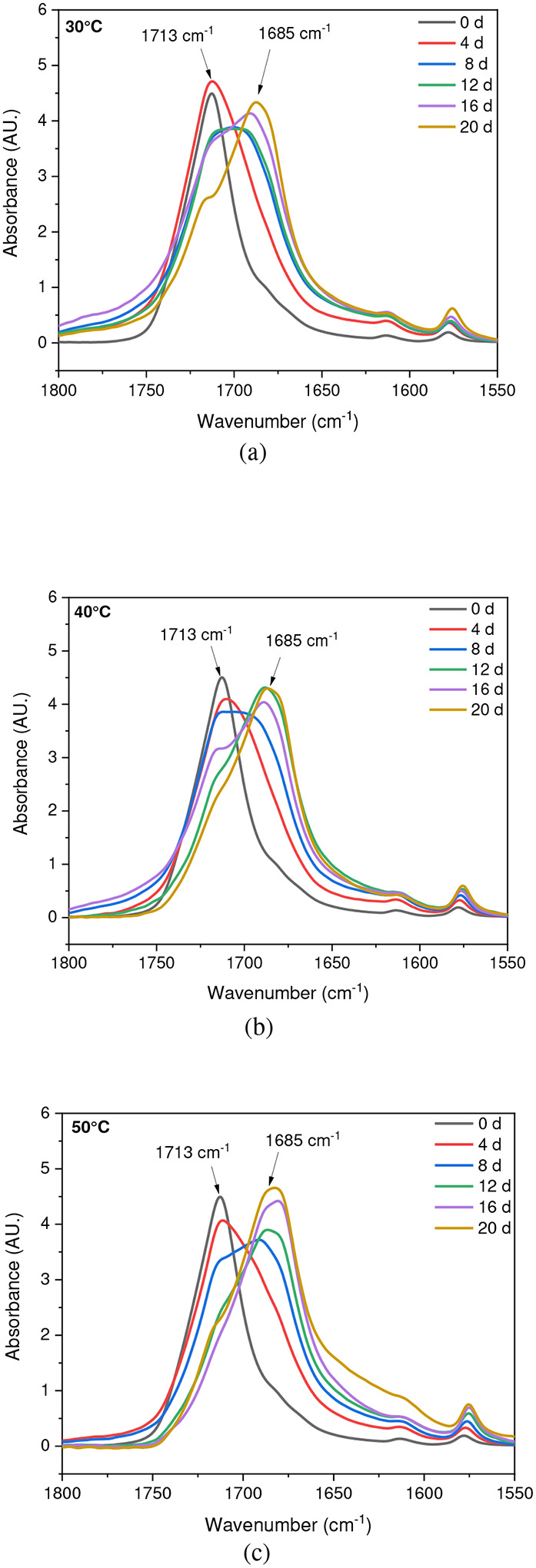
ATR-FTIR results of UV-exposed outer surfaces in saturated humidity conditions at (a) 30 °C, (b) 40 °C, and (c) 50 °C. The arrows indicate the location of 1713 cm^−1^ and 1685 cm^−1^ IR bands in the spectrum

**Fig. 7:**
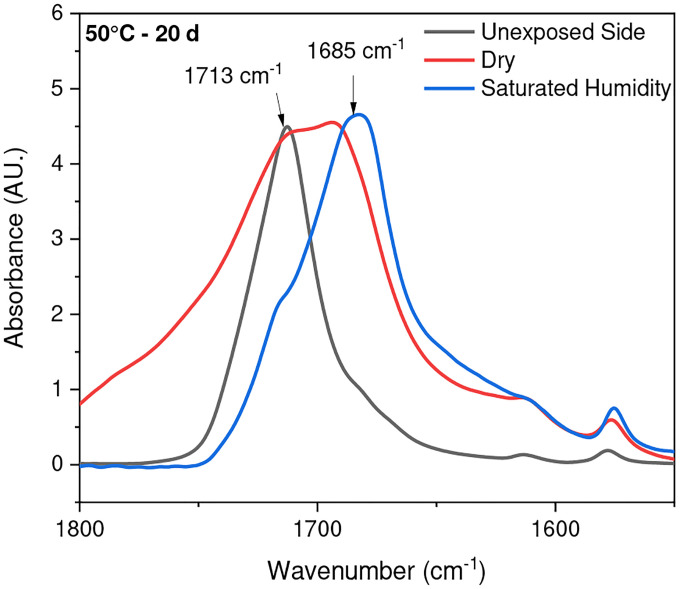
ATR-FTIR results of UV-exposed outer surfaces at 20 days exposure times in both saturated humidity and dry conditions at temperatures of 50 °C. The arrows indicate the location of 1713 cm ^−1^ and 1685 cm^−1^ IR bands in the spectrum

The stretching vibrations -(C=O) of the ester structure in PET are localized at 1713 cm^−1^ and possess different symmetry planes than the aromatic rings, allowing the formation of several structures [61]. As mentioned in the Data Analysis subsection of the Methods and Materials section, the band located at 1410 cm^−1^, attributed to phenyl ring vibration (C-H bend coupled with ring C-C stretch), serves as a reference band due to its insensitivity to orientation and conformation changes [42], [58]. Furthermore, one of the degradation products in PET is the formation of carboxylic acid, evident at a peak of 1685 cm^−1^ [41], [43]. Samples were exposed to full radiation under saturated humidity (i.e., ≥ 95 % relative humidity) and dry conditions (i.e., ≤ 5 % relative humidity) at temperatures of 30 °C, 40 °C, and 50 °C. The results corresponding to Fig. 5 and 6 demonstrate a decrease in the ester bond at peak 1713 cm^−1^ and an increase in the formation of carboxylic acid at peak 1685 cm^−1^, similar to other studies [41], [42], [58], [67], [69], [70]. Also, the results indicated that a saturated humidity (i.e., ≥ 95 % relative humidity) environment led to faster degradation at all three temperatures compared to dry conditions (i.e., ≤ 5 % relative humidity), attributed to promoting the hydrolysis process in the degradation pathway (Fig. 7).

**Fig. 8:**
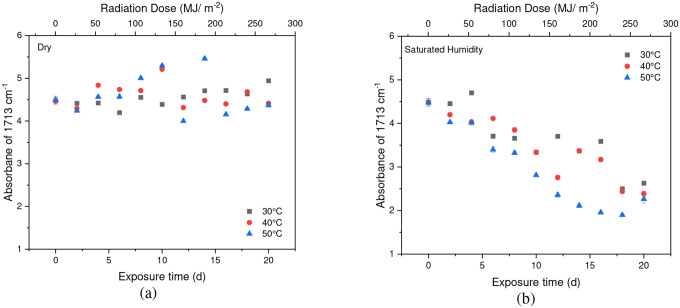
The result of 1713 cm^−1^ cleavage peak in (a) dry (b) saturated humidity conditions at various temperatures (30 °C, 40 °C, and 50 °C). Data points indicate the mean, and the error bars present the standard deviation values (n = 33)

**Fig. 9:**
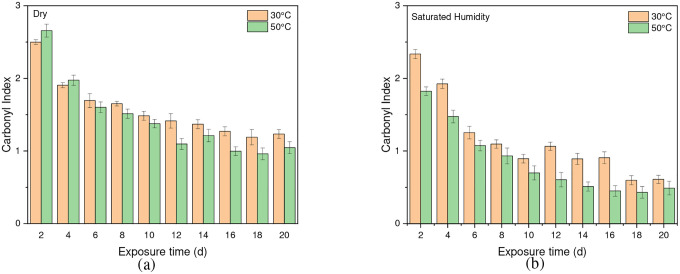
The *Carbonyl Index* for PET as a function of exposure time in (a) dry and (b) saturated humidity conditions at 30 °C and 50 °C. Data points indicate the mean, and the error bars present the standard deviation values (n = 33)

Chain scission occurs when PET is exposed to radiation in the ultraviolet (UV) region and a saturated humidity environment, primarily due to Norrish type I and type II reactions that involve radicals formed in the degradation process. Norrish type I mainly consists of the formation of radicals during ester bond cleavage, whereas Norrish type II encompasses intramolecular reactions in which a hydrogen atom is abstracted due to photolysis, resulting in polymer chains terminating in a carboxylic acid and a vinyl group [46], [61], [67], [68], [71]. The presence of humidity and full-intensity UV radiation conditions led to an apparent reduction in the ester bond at the peak of 1713 cm^−1^ (Fig. 8a). However, this result indicates that ester bond cleavage did not occur under dry UV conditions even with increasing radiation dose, indicates that ester bond cleavage is negligible under dry conditions (Fig. 8b). IR spectroscopy is a convenient and concise method for characterizing the degree of hydrolysis, and the ester/acid ratio can serve as a reliable indicator of chain scission in PET, allowing for the quantification of PET degradation. The results illustrate that the decrease in peak 1713 cm^−1^ in saturated humidity conditions is more pronounced than in dry conditions, indicating that humidity plays a significant role in the chain scission of the vinyl-ester bond in the degradation pathway, regardless of temperature. The *Carbonyl Index* also decreased with increasing exposure time, as depicted in Fig. 9.

### Effect of Temperature

**Fig. 10:**
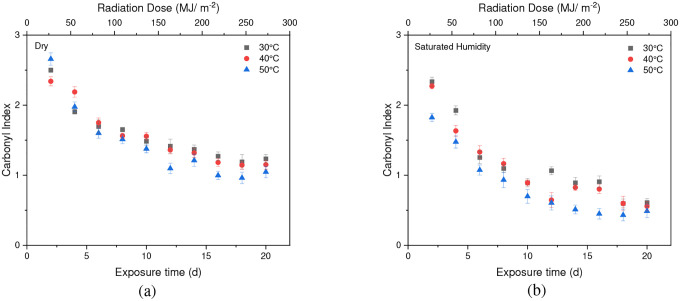
Comparing the cleavage of ester bonds at 30 °C, 40 °C, and 50 °C under both (a) dry and (b) saturated humidity conditions with the full intensity of UV radiation. Data points indicate the mean, and the error bars present the standard deviation values (n = 33)

Temperature is another significant factor influencing the cleavage of the ester bond at the 1713 cm^−1^ peak and the conformation of carboxylic acid at the 1685 cm^−1^ peak. As shown in Fig. 10, the *Carbonyl Index* at 50 °C is slightly lower than that at 30 °C in both dry and saturated humidity conditions. However, there is no discernible difference in the *Carbonyl Index* between 40 °C and 30 °C. Moreover, humidity at higher temperatures significantly initiates the degradation of PET compared to dry conditions.

**Fig. 11:**
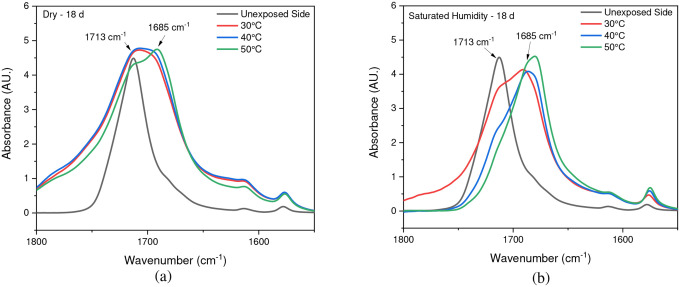
Formation of carboxylic acid at 30°C, 40°C, and 50°C under both (a) dry and (b) saturated humidity conditions with full-intensity UV radiation after 18 days of exposure time. The arrows indicate the location of 1713 cm ^−1^ and 1685 cm ^−1^ IR bands in the spectrum

**Fig. 12:**
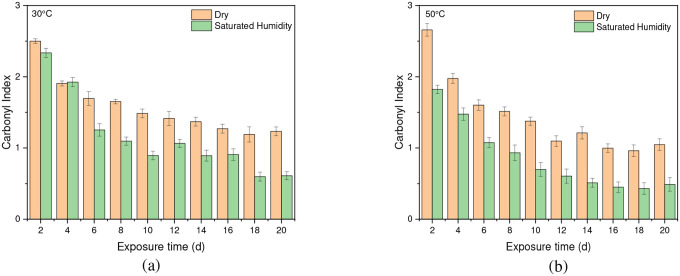
Comparing the cleavage of ester bonds at (a) 30 °C and (b) 50 °C under both dry and saturated humidity conditions with full-intensity UV radiation. Data points indicate the mean, and the error bars present the standard deviation values (n = 33)

One study has indicated that when PET undergoes thermal treatment, its ester bonds break randomly in a process involving a six-membered cyclic transition state. This initial breakdown (pyrolysis) produces vinyl esters and carboxylic acids. These primary products then react further (secondary reactions) to form various byproducts, including carbon monoxide (CO), carbon dioxide (CO_2_), acetaldehyde, aromatic acids, and their corresponding vinyl esters [71]. Fig. 11 indicates that the formation of a specific chemical compound, carboxylic acid, at peak 1685 cm^−1^ is higher at 50 °C compared to lower temperatures, 30 °C, and 40 °C. This means that the rate of formation of carboxylic acid produced increases at a higher temperature. Water absorption in PET does increase with temperature, but this alone does not guarantee faster degradation. The rate of PET hydrolysis depends on several factors, including temperature, enzyme concentration, pH, and PET type. While higher temperatures can accelerate hydrolysis [17]. The results indicate that a higher temperature (50 °C) initiates the degradation process sooner than a lower temperature (30 °C). Fig. 12 shows that at higher temperatures, the molecules have more kinetic energy, vibrating more intensely and increasing the likelihood of bond breaking. This leads to faster initiation of the degradation process [72], [73].

### Summary

This study assesses the influence of UV radiation, humidity, and temperature on PET degradation, specifically examining changes in chemical properties and concentrations of chemical structures. The NIST SPHERE was utilized to accelerate the weathering process under both dry and saturated humidity conditions at temperatures ranging from 30 °C to 50 °C and exposure periods ranging from zero day to 20 days. The data reveal that UV radiation is the most important factor, resulting in the decrease of the ester bond at peak 1713 cm^−1^ and the formation of the carbonyl group at peak 1685 cm^−1^. However, the unexposed surface of PET pieces displayed no chemical changes, even under conditions of saturated humidity and various temperatures of 30 °C, 40 °C to 50 °C. The findings indicate that humidity is a key factor in promoting the oxidative breakdown of ester bonds at the 1713 cm^−1^ peak, and this cleavage intensifies with increasing exposure times and rising temperatures. Nevertheless, the cleavage of the ester bond at peak 1713 cm^−1^ did not occur in the dry condition at various temperatures. Additionally, the formation of carboxylic acid at the 1685 cm^−1^ peak increases in saturated humidity conditions with higher temperatures and longer exposure times. Therefore, humidity is an essential factor in the breakage of easter bonds in the degradation process. Furthermore, temperature emerges as another factor influencing environmental aging. Results from FTIR indicate increased formation of carboxylic acid at 50 °C compared to 30 °C and 40 °C; this suggests that temperature slightly contributes to the aging process. These conclusions are specific to the tested water bottles and may not necessarily apply to other materials.

## Data Availability

The raw/processed data required to reproduce these findings can be shared upon request contingent upon internal approval of the National Institute of Standards and Technology.
